# Plastic female choice to optimally balance (k)in- and out-breeding in a predatory mite

**DOI:** 10.1038/s41598-020-64793-9

**Published:** 2020-05-12

**Authors:** Peter Schausberger, Demet Çekin

**Affiliations:** 1 Department of Behavioral and Cognitive Biology, University of Vienna, Althanstrasse 14, 1090 Vienna, Austria; 2 Group of Arthropod Ecology and Behavior, Department of Crop Sciences, University of Natural Resources and Life Sciences, Gregor Mendelstrasse 33, 1180 Vienna, Austria; 3 Sugadaira Research Station, Mountain Science Centre, University of Tsukuba, Ueda, Nagano, Japan

**Keywords:** Ecology, Behavioural ecology

## Abstract

Both close inbreeding and extreme outbreeding may negatively affect direct fitness. Optimal outbreeding theory suggests that females should preferentially mate with distantly related males. (K)in breeding theory suggests that, at similar direct fitness costs of close inbreeding and extreme outbreeding, females should prefer close kin to non-kin. Empirical evidence of plastic female choice for an optimal balance between close inbreeding and extreme outbreeding remains elusive. We tested the combined predictions of optimal outbreeding and (k)in breeding theories in predatory mites *Phytoseiulus persimilis* from two origins, Sicily and Greece, which suffer from both close inbreeding and extreme outbreeding depression. In three separate experiments, virgin females were presented binary choices between familiar and unfamiliar brothers, and between familiar/unfamiliar brothers and distant kin or non-kin. Females of Greece but not Sicily preferred unfamiliar to familiar brothers. Females of both origins preferred distant kin to unfamiliar and familiar brothers but preferred unfamiliar brothers to non-kin. Females of Sicily but not Greece preferred familiar brothers to non-kin. The suggested kin recognition mechanisms are phenotype matching and direct familiarity, with finer-tuned recognition abilities of Greece females. Overall, our experiments suggest that flexible mate choice by *P. persimilis* females allows optimally balancing inclusive fitness trade-offs.

## Introduction

Both close inbreeding and extreme outbreeding may negatively affect fitness. Close inbreeding commonly decreases direct fitness, for example, by reducing survival, reproduction, competitiveness and/or stress tolerance^1–3^. Outbreeding is often advantageous to close inbreeding but its benefits depend on the level of genetic distance between mates and the choice options. Extreme outbreeding may be detrimental to direct fitness for various reasons such as underdominance (i.e. heterozygote disadvantage), breaking up advantageous co-adapted gene complexes or maladaptation^4–7^. Accordingly, optimal outbreeding theory^4^ states that intermediate levels of genetic relatedness between mates should yield the highest fitness gains. However, an alternative theory of mate choice, (k)in breeding theory^8,9^, recognizes that inbreeding entails kin-selected indirect fitness gains^10,11^, due to allowing genetically related males to sire offspring and increased genetic relatedness of offspring, which may counterbalance or even outweigh direct fitness costs^8,9,12^. Synthesis of optimal outbreeding theory^4^ and (k)in breeding theory^8,9^ suggests that animals should optimally balance mate choice between kin and non-kin, in dependence of the choice options and the associated benefit-cost trade-offs in direct and indirect fitness^13^.

Optimally balancing mate choice between kin and non-kin requires kin recognition abilities^14–16^. Kin recognition is a prime mechanism allowing to either avoid or prefer inbreeding across animals; other reported mechanisms include dispersal, polyandry, delayed maturation, and/or reproductive suppression^17^. For example, individuals of many group-living species use social familiarity based on prior association as proxy of close kin and prefer unfamiliar mates to avoid close inbreeding and loss of direct fitness (for review^17,18^; for naked mole rats^19^). In contrast, inbreeding preference, likely to increase indirect fitness gains, has been observed in solitarily living harvest mice^20^, group-living lizards^21^, cichlids^22^, and fruit flies^23^. While behavioral avoidance or preference of inbreeding is empirically well documented, evidence of optimally balancing in- and out-breeding by selective mate choice is rare. Bateson’s (1982)^24^ seminal study on quail is a classic example of optimal mate choice balance, using a fivefold choice experimental design. However, behavioral studies on mate choice based on genetic relatedness often lack supporting evidence of the adaptive value of in- and out-breeding in the animal in question. A notable exception is Keane (1990)^25^, who showed, by using a fourfold choice experimental design, that mate choice of white-footed mice aligns with in- and out-breeding depression effects. Moreover, in varying choice scenarios, animals should flexibly adjust in- and out-breeding preference according to the available options, but adaptive flexible female choice has been rarely assessed. Richard *et al*.^26^ observed conditional inbreeding in dependence of age and corpulence in a semi-natural population of common lizards.

Here we addressed the combined predictions of optimal outbreeding^9^ and (k)in breeding^7,8^ theories in the plant-inhabiting predatory mite *Phytoseiulus persimilis*. These predators suffer from both close inbreeding and extreme outbreeding depression^27^. Using the same two populations (Sicily and Greece) as involved in the present study, Atalay and Schausberger^27^ showed that both sib-mated females and females mated to non-kin from the other population have ^~^25% lower lifetime reproductive success (LRS) than females mated to distant kin, i.e. mates from the same population but more distantly related than siblings. *Phytoseiulus persimilis* reproduce sexually, with males actively searching for females but, after mate encounter, females controlling whether copulation takes place or not^28,29^. The female mating system is characterized as monandry to low level of polyandry, with one or two mates per female per life, while males are highly polygynous^28,29^. Due to having only one or two mates, optimal female choice is central to LRS. *Phytoseiulus persimilis* is a specialized predator of herbivorous spider mites such as *Tetranychus urticae*^30,31^, lives in groups and possesses kin recognition abilities, which are mechanistically based on prior association^32–37^. Group-living by the predators is a consequence of the patchy distribution of the spider mites on their host plants^30,38^ and mutual attraction^37^. Spider mite-infested plants may be colonized by single females or multiple females from different origins^33,34^. Short-range dispersal happens by ambulation but long-range dispersal is largely passive by using wind currents^38^. Thus, potential mates may be related or unrelated and familiar or unfamiliar, setting the stage for the evolution of kin recognition abilities. Depending on the behavioral and ecological contexts, the predators may use direct familiarity, allowing recognizing individuals met before, and indirect familiarity (dubbed phenotype matching), allowing also recognizing unfamiliar individuals bearing the same cue as those met before^39,40^, to discriminate kin and non-kin. We hypothesized that *P. persimilis* females should flexibly adjust mate choice depending on the choice options and the associated trade-offs in direct and indirect fitness^27^. They should optimally balance mate choice by avoiding sib-mating when the alternative is distant kin but should prefer sib-mating when the alternative is non-kin.

## Methods

### Experimental animals, population origins and rearing

Experimental individuals derived from two distinct laboratory-reared populations of *P. persimilis*. The laboratory populations were each founded by 50 to 100 specimens, field-collected in either Sicily or Greece, where *P. persimilis* is native^41^. The populations were maintained in the laboratory for about 6 to 7 yrs before use in the experiments^27^. Wide geographic separation suggests lacking or negligible genetic exchange between *P. persimilis* from Sicily and Greece. In the laboratory, the predators were reared in heaps of spider mite-infested bean leaves on artificial arenas consisting of plastic tiles (15 × 15 × 0.2 cm), which rested on water-saturated foam cuboids in plastic boxes (20 × 20 × 6 cm) half-filled with water. Spider-mite infested bean leaves were added onto arenas in 2- to 3-day intervals^28^.

Two-spotted spider mites, *Tetranychus urticae*, were reared on whole bean plants *Phaseolus vulgaris*, grown at 23 ± 2 °C and 16:8 h L:D photoperiod. For both the pre-experimental and experimental procedures, the spider mites were either manually brushed from infested leaves onto fresh detached leaves, using a soft-haired paint brush, or were first brushed onto glass plates, using a mite brushing machine (BioQuip, CA, USA), and were then picked up and placed onto detached leaves or into the experimental acrylic cages using a fine red marten’s hair brush.

All rearing and experimental units were kept in a climate chamber at 25 ± 1 °C, 65 ± 5% RH and 16:8 h L:D photoperiod.

### Pre-experimental procedures

To generate experimental animals we used detached bean leaf arenas infested by spider mites and closed acrylic cages. Each leaf arena consisted of a bean leaf placed upside down on a water-saturated foam cuboid (8 × 8 × 4 cm) covered with moist filter paper. The foam cuboid was kept in a small plastic box (10 × 10 × 6 cm) half-filled with water; the edges of the leaves were covered by strips of moist tissue paper to prevent the mites from escaping. Acrylic cages consisted of cylindrical cells of 15 mm diameter and 3 mm height, laser-cut into rectangular acrylic plates, closed with fine gauze at the bottom and a microscope slide on the upper side^42^.

To obtain even-aged eggs of *P. persimilis*, giving rise to females whose offspring were used in the experiments, gravid females were randomly withdrawn from the population rearing units and placed on detached leaf arenas infested by *T. urticae*. Eggs were daily collected with a fine brush, transferred to new leaf arenas infested by *T. urticae*, and reared there until the deutonymphal stage, which is the last premature stage. Female deutonymphs were singly isolated inside acrylic cages that had been previously loaded with mixed spider mite stages, serving as prey for the predators, and cages were stored upside down on a grid inside plastic boxes, the bottom of which was covered by water to maintain elevated humidity inside the cages. After reaching adulthood inside the cages, each female was paired with a male randomly taken from its respective population. Offspring of these females were used as experimental individuals. Newly laid eggs were collected once per day and stored inside separate acrylic cages in the fridge at 8 °C. When approximately 20 to 25 eggs, ≤24 h old, were available per female, all her eggs were taken out from the fridge, split into two groups, each consisting of 10 to 15 eggs, and each group placed onto a separate detached leaf arena infested by spider mites. Siblings were reared to the deutonymphal stage within their group allowing to familiarize with siblings of this group but remaining unfamiliar with siblings of the other group. Splitting the sibling eggs into two separate groups thus allowed generating familiar and unfamiliar siblings. Deutonymphs were picked up from the leaves and singly isolated inside acrylic cages, which had been previously loaded with mixed spider mite stages, until reaching adulthood. After reaching adulthood, the individuals were sexed and ready for use in the experiments. Before use in experiments, adult males were marked with red or blue water color dots (Jolly; Brevillier Urban & Sachs, Vienna, AT) on their dorsal shields to make them distinguishable from the alternative mate option in female choice tests. Red and blue were randomly assigned to the two males presented to a female.

### Experimental procedures

We conducted three choice experiments to determine female mating preference in dependence of familiarity and genetic relatedness of the male mate options (Table 1). To assess female choice, single virgin females from both populations, Sicily and Greece, were presented two virgin males differing in familiarity and/or degree of genetic relatedness to the female. Male mate options had one of three degrees of genetic relatedness to the female, close kin (sibling), distant kin (same population), or non-kin (different population). Due to long-term rearing history in the laboratory (closed populations without immigration and emigration) we assumed approximate coefficients of relatedness (r) of r = 0.5 for siblings, 0 < r < 0.5 for distant kin, and r = 0 for non-kin. Siblings were either familiar or unfamiliar to the female, non-siblings were always unfamiliar (Table 1). To start a choice experiment, the two males were placed inside an acrylic cage (construction as described in pre-experimental procedures) containing spider mites as prey and one virgin female was added. Cages were monitored in 20 min intervals, using a stereo-microscope, until the first successful mate choice by the female occurred. Female choice was scored successful when one of the two males was clinging to the female from beneath in the venter to venter position for copulation^43^. This experimental setup and protocol minimized any potential influence of male-male competition and pinpointed female choice^28,29^. The first experiment aimed at finding out whether *P. persimilis* females are able to use direct familiarity (prior association) to recognize siblings and avoid mating with them when given a choice between a familiar and an unfamiliar sibling (Table 1). The second experiment aimed at determining whether *P. persimilis* females are able to discriminate siblings from distant kin (individuals from the same population but no siblings). Siblings were either familiar or unfamiliar to the female (Table 1). The third experiment aimed at determining whether *P. persimilis* females are able to discriminate siblings from non-kin (individuals from the other population). Siblings were either familiar or unfamiliar to the female (Table 1). In each experiment, each female, male and cage were used only once. Every female used in the experiments mated with one or the other male, no female was discarded from analysis.

**Table 1:** Experimental design used to assess female choice. Single females from two populations, Sicily and Greece, were offered two males differing in relatedness and/or familiarity to the female (F for familiar, UF for unfamiliar). Distant kin males came from the same population as the female but were no siblings, non-kin males came from the other population. All females and males were virgin and used only once in experiments.

Experiment	Female origin (replicates)	Male 1	Male 2
1	Sicily (17); Greece (15)	Sibling (F)	Sibling (UF)
2	Sicily (16); Greece (19)	Sibling (F)	Distant kin (UF)
	Sicily (16); Greece (16)	Sibling (UF)	Distant kin (UF)
3	Sicily (16); Greece (15)	Sibling (F)	Non-kin (UF)
	Sicily (15); Greece (16)	Sibling (UF)	Non-kin (UF)

### Statistical analyses

Statistical analysis was carried out using IBM SPSS 23 (IBM, Armonk, NY, USA). Separate generalized linear models (GLM, binary logistic distribution) were run for each experiment and each choice scenario (a given combination of one female and two males; Table 1) within experiments; intercepts were used as indicators of preference. In the first step, to verify unbiasedness of the experimental setup, we assessed color choice (red vs. blue) using population origin of the females as independent variable. In the second step, we assessed familiarity choice (familiar vs. unfamiliar) or relatedness choice (sibling vs. distant kin or sibling vs. non-kin) using population origin of the females as independent variable. In the third step, we compared familiarity choice (familiar vs. unfamiliar) or relatedness choice (sibling vs. distant kin or sibling vs. non-kin) within each population. Alternative GLMs (not shown here) by skipping step 1 and include color as independent variable in steps 2 and 3 revealed qualitatively similar results.

## Results

In no experiment and in no choice scenario did females of either population, Sicily and Greece, show a biased preference for red or blue males (GLM; intercept χ_1_^2^ < 1.116, P > 0.29; population origin of female χ_1_^2^ < 0.425, P > 0.51 for any scenario).

**Figure 1:**
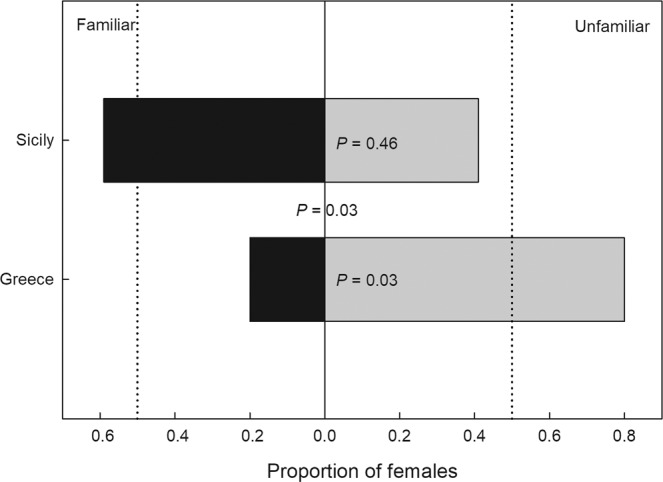
Female choice between familiar and unfamiliar siblings. Mate preference of virgin predatory mite females *Phytoseiulus persimilis* from Sicily and Greece presented a binary choice of a familiar brother and an unfamiliar brother. Dotted vertical lines indicate the expected proportions at no preference. P-levels inside bars refer to GLM within each population and choice scenario, assuming random choice; the P-level between bars refers to GLM between the two populations, assuming same choice. N = 15 (Greece) and 17 (Sicily).

The first experiment examined the use of direct familiarity to recognize siblings. Choice between familiar and unfamiliar siblings differed between females from Sicily and Greece (χ_1_^2^ = 4.606, P = 0.03). While Sicily females did not discriminate familiar and unfamiliar siblings (χ_1_^2^ = 0.524, P = 0.46), Greece females mated preferentially with unfamiliar siblings (χ_1_^2^ = 4.612, P = 0.03) (Fig. 1).

**Figure 2:**
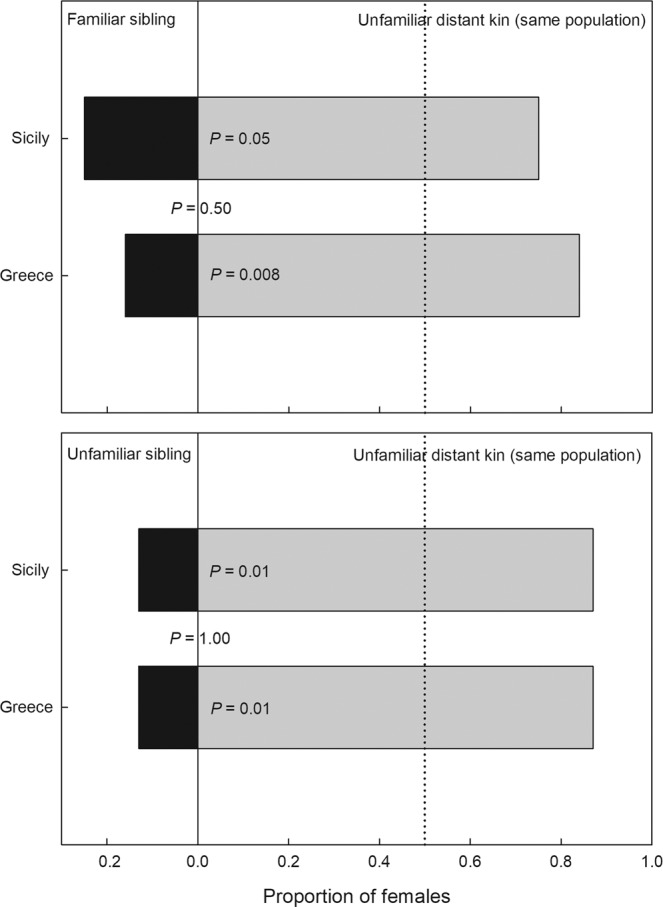
Female choice between siblings and distant kin. Mate preference of virgin predatory mite females *Phytoseiulus persimilis* from Sicily and Greece presented a binary choice of a familiar or an unfamiliar brother and a distant kin male (coming from the same population but no siblings). The dotted vertical line indicates the expected proportion at no preference. P-levels inside bars refer to GLM within each population and choice scenario, assuming random choice; the P-level between bars refers to GLM between the two populations, assuming same choice. N = 19 (Greece) and 16 (Sicily) for familiar sibling vs. unfamiliar distant kin and 16 (Greece) and 16 (Sicily) for unfamiliar sibling vs. unfamiliar distant kin.

The second experiment examined discrimination between siblings and distant kin. Choice between familiar or unfamiliar siblings vs. unfamiliar distant kin did not differ between females from Sicily and Greece (familiar siblings: χ_1_^2^ = 0.454, P = 0.50; unfamiliar siblings: χ_1_^2^ = 0.000, P = 1.00). Females from both populations preferentially mated with unfamiliar distant kin (familiar siblings Sicily: χ_1_^2^ = 3.621, P = 0.05, Greece: χ_1_^2^ = 7.079, P = 0.008; unfamiliar siblings Sicily: χ_1_^2^ = 6.626, P = 0.01, Greece χ_1_^2^ = 6.626, P = 0.01; Fig. 2).

**Figure 3:**
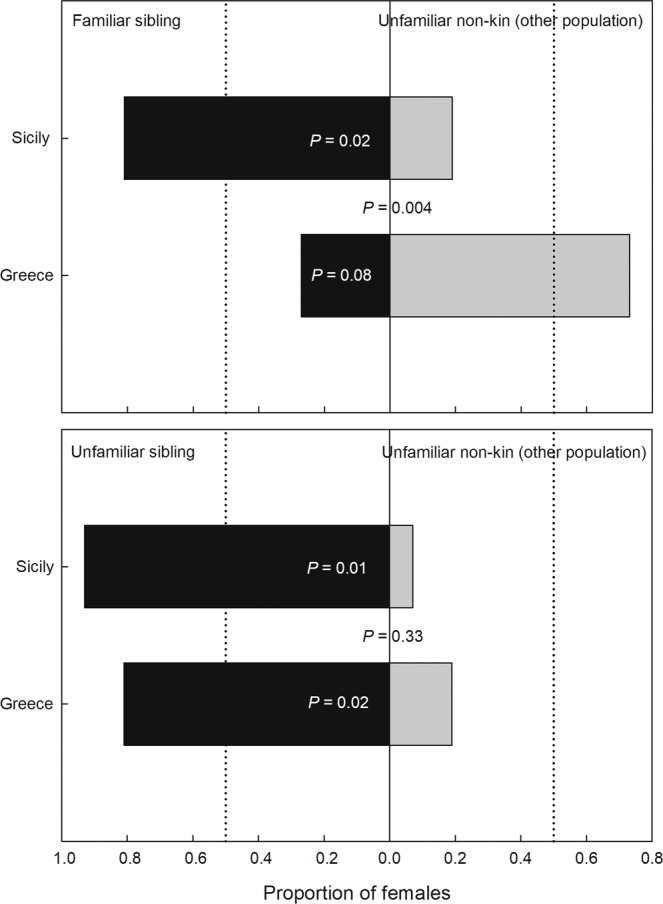
Female choice between siblings and non-kin. Mate preference of virgin predatory mite females *Phytoseiulus persimilis* from Sicily and Greece presented a binary choice of a familiar or an unfamiliar brother and a non-kin male (coming from another population). Dotted vertical lines indicate the expected proportions at no preference. P-levels inside bars refer to GLM within each population and choice scenario, assuming random choice; the P-level between bars refers to GLM between the two populations, assuming same choice. N = 15 (Greece) and 16 (Sicily) for familiar sibling vs. unfamiliar non-kin and 16 (Greece) and 15 (Sicily) for unfamiliar sibling vs. unfamiliar non-kin.

The third experiment examined discrimination between siblings and non-kin. Choice between familiar or unfamiliar siblings vs. unfamiliar non-kin differed between females from Sicily and Greece when the siblings were familiar (χ_1_^2^ = 8.174, P = 0.004) but did not differ when the siblings were unfamiliar (χ_1_^2^ = 0.928, P = 0.33). While Greece females did not discriminate familiar siblings from unfamiliar non-kin (χ_1_^2^ = 3.002, P = 0.08), Sicily females mated preferentially with familiar siblings (χ_1_^2^ = 5.241, P = 0.02). Females from both populations preferentially mated with unfamiliar siblings when the alternative option was unfamiliar non-kin (Sicily: χ_1_^2^ = 6.500, P = 0.01; Greece: χ_1_^2^ = 5.241, P = 0.02; Fig. 3).

## Discussion

The results of our study suggest that females of the predatory mite *P. persimilis* optimally balance mate choice in dependence of the choice options and associated inclusive fitness trade-offs^4,5,27^. Such behavior meets the combined predictions of optimal (k)in- and out-breeding theories^7–9^. Overall, females, which are controlling whether copulation takes place or not^28,29^, preferred distant kin to close kin (experiment 2) but preferred close kin to non-kin (experiment 3). Flexible female choice by *P. persimilis* is adaptive because distant kin provide higher inclusive fitness benefits than close kin (in relative comparison, direct fitness gains outweighing indirect fitness loss) whereas close kin provide higher inclusive fitness benefits than non-kin (in relative comparison, additional indirect fitness gain at similar direct fitness trade-offs)^27^. Lacking preference of Greece females presented a binary choice of familiar sibling mates and unfamiliar non-kin mates could have been due to lower average relatedness, and associated lower inbreeding level and lower achievable indirect fitness gains, in the Greece population than the Sicily population. This assumption requires further scrutiny.

Theoretically, the cues used for non-kin mate (extreme outbreeding) avoidance may represent kin-selected labels or be epiphenomena of diverging sexually-selected signals. For the following reasons we favor the explanation of kin-selected labels in our experimental system. The predatory mites *P. persimilis* have been shown to discriminate kin and non-kin in mate choice^33^ and various other, non-sexual, contexts such as cannibalism and group-living^32,34–37^. Individuals of the two populations used here, Sicily and Greece, are reproductively perfectly compatible and readily mate with each other under no-choice^27^. Therefore, we argue that the females most likely based their mating decisions on comparatively evaluating the level of similarity between cues from close kin versus cues from distant kin and non-kin.

Experiments 1 and 2 revealed that females from Greece and Sicily differ in the use of kin recognition mechanisms, with Greece females showing finer-tuned kin discrimination than Sicily females. Greece females were able to use both direct familiarity (evident from experiment 1) and phenotype matching (indirect familiarity; evident from experiments 2 and 3), whereas Sicily females only used phenotype matching (evident from experiments 1 to 3) to assess the mate options. Sicily females apparently familiarized with siblings during development, formed a generalized template of siblings and then discriminated siblings from distant kin and non-kin by phenotype matching^39,40^. However, use of a generalized template of siblings did not allow them to discriminate familiar and unfamiliar siblings (experiment 1). In contrast, Greece females could discriminate familiar and unfamiliar siblings. Accordingly, direct familiarity was a stronger indicator of close kin than indirect familiarity for Greece but not Sicily females. The observed inter-population difference may be due to evolution of finer-grained recognition abilities in the Greece than Sicily population and/or to a higher level of inbreeding in the Sicily than Greece population. Both populations had been closed and inbred in the laboratory for several years before the experiment, with bottleneck events likely differing between the two populations. Higher average level of genetic relatedness in the Sicily population may have decreased label variability, resulting in lower acceptance thresholds of Sicily females^44,45^.

In conclusion, many studies documented that kin recognition allows avoidance or preference of inbreeding^17^, both in vertebrates and invertebrates, such as common voles^46^, field crickets^47^, ants^48^, termites^49^, cichlids^22^, or lizards^21^. In contrast, few studies tested the hypothesis of balanced in- and out-breeding by kin recognition linked to documented in- and out-breeding depression effects in the study animals^25^. Our study provides an example of animals flexibly basing female choice on inclusive fitness trade-offs of in- and out-breeding^27,50^ in dependence of the binary mate options. Future studies should experimentally address presumable transitivity in mate choice^51^ by offering females multiple mate options ranging from close kin over various degrees of more distant kin to non-kin from various populations differing in genetic distance and achievable fitness.

## Data Availability

The datasets generated and/or analyzed during the current study are available from the corresponding author on reasonable request.
